# Defining the *In Vivo* Phenotype of Artemisinin-Resistant *Falciparum* Malaria: A Modelling Approach

**DOI:** 10.1371/journal.pmed.1001823

**Published:** 2015-04-28

**Authors:** Lisa J. White, Jennifer A. Flegg, Aung Pyae Phyo, Ja Hser Wiladpai-ngern, Delia Bethell, Christopher Plowe, Tim Anderson, Standwell Nkhoma, Shalini Nair, Rupam Tripura, Kasia Stepniewska, Wirichada Pan-Ngum, Kamolrat Silamut, Ben S. Cooper, Yoel Lubell, Elizabeth A. Ashley, Chea Nguon, François Nosten, Nicholas J. White, Arjen M. Dondorp

**Affiliations:** 1 Centre for Tropical Medicine, Nuffield Department of Medicine, University of Oxford, Oxford, United Kingdom; 2 Mahidol-Oxford Tropical Medicine Research Unit, Faculty of Tropical Medicine, Mahidol University, Bangkok, Thailand; 3 Worldwide Antimalarial Resistance Network, Oxford University, Oxford, United Kingdom; 4 Shoklo Malaria Research Unit, Mahidol-Oxford Tropical Medicine Research Unit, Faculty of Tropical Medicine, Mahidol University, Mae Sot, Thailand Armed Forces Research Institute of Medical Sciences, Bangkok, Thailand; 5 Howard Hughes Medical Institute/Center for Vaccine Development, University of Maryland School of Medicine, Baltimore, Maryland, United States of America; 6 Department of Genetics, Texas Biomedical Research Institute, San Antonio, Texas, United States of America; 7 National Malaria Center, Ministry of Health, Phnom Penh, Cambodia; Swiss Tropical & Public Health Institute, SWITZERLAND

## Abstract

**Background:**

Artemisinin-resistant *falciparum* malaria has emerged in Southeast Asia, posing a major threat to malaria control. It is characterised by delayed asexual-stage parasite clearance, which is the reference comparator for the molecular marker ‘Kelch 13’ and *in vitro* sensitivity tests. However, current cut-off values denoting slow clearance based on the proportion of individuals remaining parasitaemic on the third day of treatment ('day-3'), or on peripheral blood parasite half-life, are not well supported. We here explore the parasite clearance distributions in an area of artemisinin resistance with the aim refining the *in vivo* phenotypic definitions.

**Methods and Findings:**

Data from 1,518 patients on the Thai-Myanmar and Thai-Cambodian borders with parasite half-life assessments after artesunate treatment were analysed. Half-lives followed a bimodal distribution. A statistical approach was developed to infer the characteristics of the component distributions and their relative contribution to the composite mixture.

A model representing two parasite subpopulations with geometric mean (IQR) parasite half-lives of 3.0 (2.4-3.9) hours and 6.50 (5.7-7.4) hours was consistent with the data. For individual patients, the parasite half-life provided a predicted likelihood of an artemisinin-resistant infection which depends on the population prevalence of resistance in that area. Consequently, a half-life where the probability is 0.5 varied between 3.5 and 5.5 hours. Using this model, the current 'day-3' cut-off value of 10% predicts the potential presence of artemisinin-resistant infections in most but not all scenarios. These findings are relevant to the low-transmission setting of Southeast Asia. Generalisation to a high transmission setting as in regions of Sub-Saharan Africa will need additional evaluation.

**Conclusions:**

Characterisation of overlapping distributions of parasite half-lives provides quantitative insight into the relationship between parasite clearance and artemisinin resistance, as well as the predictive value of the 10% cut-off in 'day-3' parasitaemia. The findings are important for the interpretation of *in vitro* sensitivity tests and molecular markers for artemisinin resistance and for contextualising the ‘day 3’ threshold to account for initial parasitaemia and sample size.

## Abstract 2

Lisa White and colleagues explore parasite clearance distributions in an area of artemisinin resistance with the aim of refining the phenotypic definition of resistance.

## Editors' Summary

**Background:**

Artemisinin and its derivatives are powerful medicines that can quickly reduce the number of *Plasmodium* parasites in the blood of patients with malaria. Artemisinin combination therapies (ACTs) are recommended by the WHO as the first-line treatment for uncomplicated *Plasmodium falciparum* malaria. Expanding access to ACTs has been a key contributor to the recent success in reducing the global malaria burden. Several hundred million ACT treatment courses are currently distributed in malaria-endemic countries every year. However, recent findings of artemisinin-resistant malaria parasites have alarmed the global health community. According to the WHO, as of February 2015, artemisinin resistance had been confirmed in five countries in Southeast Asia, and its spread is seen only as a question of time by many experts. Such spread—or independent emergence of artemisinin resistance—in other parts of the world would pose a major health crisis because there are currently no other antimalarial drugs that are as effective and well-tolerated as ACTs.

**Why Was This Study Done?:**

Surveillance systems to detect the spread or emergence of artemisinin resistance in malaria-endemic regions are crucial so that control and elimination measures can be undertaken rapidly. Tools for surveillance include clinical studies on parasite clearance from the blood, *in vitro* parasite testing for artemisinin sensitivity in the laboratory, and assessment of molecular markers. (Tests based on molecular markers of resistance are being developed based on the 2013 discovery of artemisinin-resistance mutations in a parasite gene called *Kelch 13* that are associated with delayed parasite clearance after ACT treatment.) The best description of parasite clearance in the individual patient is the parasite half-life, which requires frequent blood sampling. Since this is difficult to do in resource-limited settings, the WHO uses the following working definition: artemisinin resistance in a population is suspected if more than 10% of patients are still carrying parasites three days after the start of ACT treatment. The researchers conducted this study to see how well this working definition matches actual data from individual patients in areas with artemisinin-resistant *P*. *falciparum* parasites.

**What Did the Researchers Do and Find?:**

The researchers used data on blood samples from 1,518 patients collected along the Thai-Myanmar and Thai-Cambodian borders (where resistance first developed) from 2001 to 2012. All patients had been treated with ACT, and the time it took for the drugs to kill half of the parasites in their blood (the parasite half-life) had been measured. When the researchers used mathematical models to analyse these parasite half-life assessments after ACT treatment, they found that a model that assumed two different parasite subpopulations best explained the actual data. One of these subpopulations—and the only one present in the early years before resistance developed—had a shorter parasite half-life of about 3 hours (equivalent to sensitive parasites that still are killed efficiently by the ACT drugs). The second population began to appear around 2008 and had a longer half-life of about 6.5 hours, equivalent to parasites that had acquired resistance to artemisinin, and consistent with measurements of Kelch 13–mutant parasites. The parasite half-life predicted the likelihood of an artemisinin-resistant infection for individual patients, but this was influenced by how common resistance was in that area: the critical half-life varied between 3.5 hours (in areas where resistance is rare) and 5.5 hours (in areas where resistance is common). This means that there is not a universal cut-off value in parasite half-life to denote an infection as ‘sensitive’ or ‘resistant’ because intermediate half-lives can be part of the lower tail end of the resistant parasite half-life distribution or the top-end of the sensitive one. The model the researchers developed can estimate the background proportion of resistant infections, and this can then be used to calculate the probability for an individual infection to belong to a resistant or sensitive parasite half-life distribution, thereby providing a more accurate assessment of resistance. Taking this into account, the researchers found that the current WHO 'day-3' cut-off value of 10% predicts the potential presence of artemisinin-resistant infections in most but not all scenarios.

**What Do these Findings Mean?:**

The findings are relevant to the situation in Southeast Asia, where malaria is less common than in so-called high-transmission areas, including much of Sub-Saharan Africa. Whether the model is useful for resistance surveillance in high-transmission areas will need further study. Nonetheless, the results suggest that a model that analyses data on parasite half-life as distributions of artemisinin-sensitive and artemisinin-resistant populations is a useful tool for surveillance of artemisinin resistance in a geographical area. The model also measures the probability that a parasite strain with a given parasite half-life is resistant to artemisinin, which should help with the evaluation of other surveillance methods, including those based on *Kelch 13* mutations and possibly other molecular markers. The WHO—recommended definition of suspected artemisinin resistance in cases where more than 10% of patients still carry parasites on the third day of treatment is useful, but, according to this study, including the initial parasite load (i.e., the number of parasites in a patient’s blood before the start of treatment) would make it more informative. In addition, as the ‘day-3’ cut-off method lacks accuracy in predicting the actual proportion of artemisinin-resistant parasites, a positive result should be followed by a more detailed assessment.

**Additional Information:**

This list of resources contains links that can be accessed when viewing the PDF on a device or via the online version of the article at http://dx.doi.org/10.1371/journal.pmed.1001823.

- The WHO issues regular updates about the status of artemisinin-resistant malaria
- The WHO provides a list of questions and answers about artemisinin-resistant malaria
- Wikipedia provides information on artemisinin (note that Wikipedia is a free online encyclopedia that anyone can edit; available in several languages)
- The Bill & Melinda Gates Foundation describes its global strategy to fight and eradicate malaria in the face of artemisinin resistance
- The Mahidol Oxford Research Unit, where several of the study’s authors work, has pages about malaria, including information about treatments and drug-resistance

## Introduction

Artemisinin combination therapies (ACTs) are currently the cornerstone of antimalarial treatment of uncomplicated *falciparum* malaria throughout the tropical world. There are no new antimalarial drugs of equivalent efficacy available to replace them, and registration of new antimalarial compounds will take at least several years. It is therefore a major threat to current global malaria control and elimination plans that artemisinin resistance has emerged in several areas of the Greater Mekong Subregion. This resistance is characterized by a markedly slower clearance of *P*. *falciparum* parasitized red blood cells from the peripheral blood [1]. Treatment of patients with a sensitive *P*. *falciparum* infection to artemisinin or an artemisinin derivative results in a 10,000 times reduction in parasitaemia per 48-hour life cycle [1]. This is reduced to a roughly 100-fold decrease in artemisinin-resistant infections [1]. This places an increased selection pressure on the ACT partner drugs, and there are now several examples where this slow clearance was a prelude to treatment failure with ACTs. Failure rates between 20% and 40% at 42 days follow-up after treatment with DHA-piperaquine are now being observed in Western Cambodia [2], and high failure rates with artesunate-mefloquine are now being observed on the Thai-Myanmar border [3]. It is feared that artemisinin-resistant parasites will spread westward and reach Africa, where the burden of *falciparum* malaria is much higher. In the last century this same route was followed by parasites resistant to chloroquine and later pyrimethamine. Chloroquine resistance is thought to have contributed to the death of millions of African children [4].

**Fig 1:**
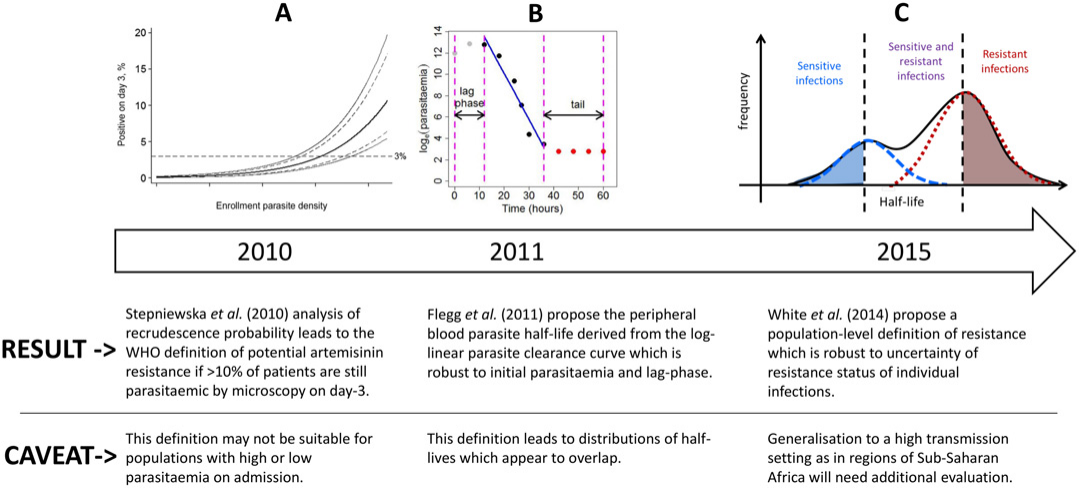
A diagram illustrating the evolution of the definition of artemisinin resistance using parasite clearance data. The first definition is based on the ‘day-3’ parasitaemia [12], the second is defined as the peripheral blood parasite half-life derived from the log-linear parasite clearance curve [10], and the third is the definition proposed in this study.

It is essential that surveillance systems are in place to document the spread or emergence of artemisinin resistance in malaria-endemic regions so that control and elimination measures can be instituted rapidly. Tools for surveillance include clinical studies on parasite clearance, artemisinin *in vitro* sensitivity testing, and assessment of molecular markers. Important progress has been made recently with the discovery of mutations in a gene on chromosome 13 coding for the propeller region of a Kelch protein. However, this does not decrease the importance of an accurate definition of the pharmacodynamic phenotype [5–7]. For instance, the interpretation of the presence of Kelch mutations in African parasites is currently an important issue [8,9]. Further exploration of the responsible genotypes as well as validation of future *in vitro* sensitivity tests will still have to use the pharmacodynamically defined resistance phenotype as a reference, which thus needs a clear definition. While the interpretation of the Kelch mutations is still under debate, this approach can be used as a reference method for resistance surveillance where suitable pharmacodynamic data are available. The parasite half-life, which requires frequent blood sampling for parasitaemia assessment, has been proposed [10] and evaluated [11] as the best description of peripheral blood parasite clearance in the individual patient. Since this is operationally difficult in resource-limited settings, the WHO working definition for ‘suspected artemisinin resistance’ in a population is if more than 10% of patients are still carrying parasites three days after the start of ACT treatment (‘day-3’ parasitaemia) [12] (Fig 1A). This general guideline is relatively easy to implement in a surveillance programme, but the predictive value for artemisinin resistance will depend on a number of variables including the initial parasitaemia, the precise timing of sampling, and sample size. Although ‘all purpose’ criteria may be simple to use, the potential caveats will need identification. This study explores the distribution characteristics of the peripheral blood parasite half-life, which is the current reference describing parasite clearance. These characteristics are explored without including genetic markers and are then validated by linking these to the parasite half-life distribution defined by presence of Kelch mutations. The results also seek to quantify the accuracy of the ‘day-3’ parasitaemia as a measure of artemisinin resistance and proposes user-friendly modifications to tailor the threshold denoting resistance to specific settings.

For individual patients, the parasite half-life is derived from the slope of the linear part of the log-transformed parasitaemia versus time profile (Fig 1B) [13,14]. The parasite half-life is the time required for the number of parasites in the peripheral blood to fall by 50%, during the log-linear clearance phase after start of antimalarial treatment [10]. A cut-off value in the parasite half-life denoting artemisinin resistance is usually taken as 5 hours, but this has not been formally validated. Many factors can affect parasite clearance apart from drug susceptibility [15], leading to a large variation in clearance rates of sensitive and resistant parasite strains and an overlap in the distributions of half-lives measured for a population of individuals (Fig 1C). Therefore the definition of artemisinin resistance began historically as a population metric (Fig 1A), then moved to a measure for each individual patient (Fig 1B), and we are now proposing once again a population approach (Fig 1C) which can in turn be used to predict a probability of being resistant for each individual infection (based on the individual’s position within a composite distribution) rather than a binary outcome (based on an individual’s half-life being above or below a cut-off value).

A method to address the contribution of host versus parasite factors was used to explore the variation in parasite half-life of which 40% to 60% can be explained by heritable genetic parasite factors [16–19]. When plotted over calendar time for specific sites, the parasite half-lives appear to be increasing [18], but it is not clear whether this increasing trend is due to a gradual shift in half-life or a mixture of subgroups defined by distinct distributions of half-lives with the proportion of individuals belonging to the long half-life subgroups increasing over time. These studies were all performed in areas of low *P*. *falciparum* transmission. In these settings genetically indistinguishable parasite strains often infect several patients enrolled in a study, and in these patients the clearance half-life of peripheral blood parasites after treatment with artemisinins cluster together. In the current study, this observation was combined with a mixture modelling approach to define the relationship between parasite half-life and resistance and to explore its implication for the current WHO definition of potential resistance within a population.

## Methods

### The Data

Our analysis included individual patient data from three published studies from Southeast Asia [16–18,20,21] which provided detailed assessments of *P*. *falciparum* parasite clearance rates and half-lives in 1,518 patients, as well as assessment of genetic similarity of parasite strains between patients. In all studies, parasitaemia was assessed in intervals of 6 hours or less after start of therapy. Parasite clearance half-life, which is the time needed to reduce parasitaemia by half, was estimated using a parasite clearance estimator developed by the Worldwide Antimalarial Resistance Network [10]. The estimator calculates parasite clearance rate based on the linear portion of the log_e_ parasitaemia-time curve, and the half-life is derived from this. The first study evaluated the changes in parasite clearance between 2001 and 2010 on the northwestern border of Thailand in patients with uncomplicated hyperparasitaemic (>4%) *falciparum* malaria treated with various oral artesunate-containing therapies [18]. Parasites were genotyped for 93 single nucleotide polymorphisms (SNPs) across the genome using an Illumina Goldengate® platform and were considered clonally identical if they were indistinguishable at all loci genotyped. A second study was performed in Pailin, Western Cambodia, in 2007–2008 [16,20]. In this study oral artesunate was given at a dose of 2 mg/kg body weight per day, for 7 days, or at a dose of 4 mg/kg for 3 days, followed by mefloquine at a dose of 25 mg/kg. Parasites were genotyped according to 18 microsatellite loci, and parasite strains were classified as identical when all loci were the same. A third study evaluating different dosing regimens of 7 days of artesunate in patients with uncomplicated *falciparum* malaria in Tasanh, Batambang Province in Western Cambodia in 2008–2009 provided parasite DNA for a genome-wide association study using an Affymetrix GeneChip® Scanner 3000 genotype platform determining 8,079 SNPs across the genome [17,21]. Parasites were classified as identical if all SNPs were the same between strains. Multi-clonal infections were excluded from the analysis.

### Mixture Models

The hypothesis we tested was whether a finite set of distinct subgroups of parasite genotypes related to varying levels of parasite half-lives could explain the observed distribution of parasite half-lives. Each subgroup (including the sensitive subgroup) can be characterised by a unimodal distribution of the clearance half-lives after treatment with ACTs. The distribution of clearance half-lives for a given location would be the weighted composite of the distributions of the underlying subgroups. A mixture modelling approach was used [22] for this purpose. The results were then applied to explore the relationship between observations of percentage positive on ‘day-3’ of treatment.

Mixture models [22] can be applied to define the set of distributions that combine to form a composite mixture distribution. The power of this approach is that information on the membership of each individual observation is not required. A finite mixture model will, for a predefined number of components (subpopulations), estimate the shapes of the distributions and relative contributions of each of the subpopulations to the observed composite population. For the half-life data in a given location and year, the number of subpopulations is unknown, therefore a series of finite mixture models from a 1-component model up to a 5-component model were fitted to each dataset using the mixtools [22] package in R [23]. The Akaike Information Criterion (AIC) [24] was calculated for each model and dataset pair and then an n-component model was compared with an (n+1)-component model by calculating the evidence ratio [24] given by $\exp\left(-\frac{\left(AIC_{n}-AIC_{n+1}\right)}{2}\right)$. An evidence ratio of less than 0.1 was chosen to favour the more parsimonious model. This condition was chosen because the sample sizes of the disaggregated datasets are relatively low (2 orders of magnitude) and this additional preference for the simpler model would reduce the risk of over-fitting [24]. Sequential models were compared using the evidence ratio. In this way, for each location and in each year, the most likely number of distributions was estimated as well as the distribution shapes and relative contributions. This process was performed twice, first assuming that the subgroups conformed to normal distributions and second assuming log-normal distributions. The R-code for the analysis in the log-normal case can be found at https://github.com/lisawhite100/PDpheno-mixture.git. The results of this analysis provided a quantitative relationship between the observed parasite clearance half-life in a *falciparum* malaria patient treated with an artemisinin and the likelihood that the infecting parasite strain is artemisinin-resistant.

The approach was then validated by comparison with the distribution of parasite half-lives of clonal parasite infections. To differentiate between host factors and parasite factors affecting parasite clearance half-life, the fact that several patients in a geographical area were infected by the same (genetically indistinguishable) parasite strain was exploited. A total of 210 parasite strains (clones as defined by the method reported in [16]), caused infections in 2 or more patients, giving a total of 634 out of 1,518 infections. The geometric mean of the parasite half-lives in infections caused by the same parasite strain (n = 210) can be assumed to represent the clearance phenotype of that parasite strain, independent of the contribution of the host. It was then explored whether the distribution of these (host independent) clone half-lives consisted of a number of distinct subgroups, rather than a continuous distribution. This should be the case if clearance half-life is more associated with the parasite rather than with the host. In a permutation analysis, the same mixture model was then applied to a set of geometric means (n = 210) obtained by randomly relabeling the 634 samples with the list of clone numbers. This process was repeated 100 times to determine whether the number of components predicted by the mixture models was related to the true labels for the clones or not. If, when clone numbers were scrambled and randomly assigned to the different infecting parasite strains, the process predicted single rather than multiple component distributions for the scrambled datasets, this would support the conclusion that the number of discrete distributions of half-lives produced by the mixture model (applied to the unscrambled data) represent the characteristic clearance half-lives of different levels of artemisinin-resistant parasite strains.

The model results were also applied to explore the current WHO-recommended cut-off value for the proportion of patients still parasitaemic after 3 days of artemisinin treatment, denoting suspected artemisinin resistance [25]. This ‘day-3’ parasitaemia measure is currently widely used as a surveillance tool for artemisinin resistance in a population with limited evidence for its suitability. The estimated sensitive and resistant distributions of half-lives were used to generate a series of simulated surveillance data comprising daily qualitative assessments of parasitaemia (peripheral blood slide positive or negative). This *in silico* experiment (see https://github.com/lisawhite100/PDpheno-mixture.git for the full R code and demonstration datasets) was carried out to explore the relationship between the expected proportion of individuals remaining parasitaemic on each day of treatment and the underlying proportion of resistant infections given a certain sample size and distribution of initial parasitaemias. Realistic distributions of the time lag between the onset of the log-linear decay of parasitaemia and the onset of treatment (as described in [10]) were included.

## Results

### The Data

**Fig 2:**
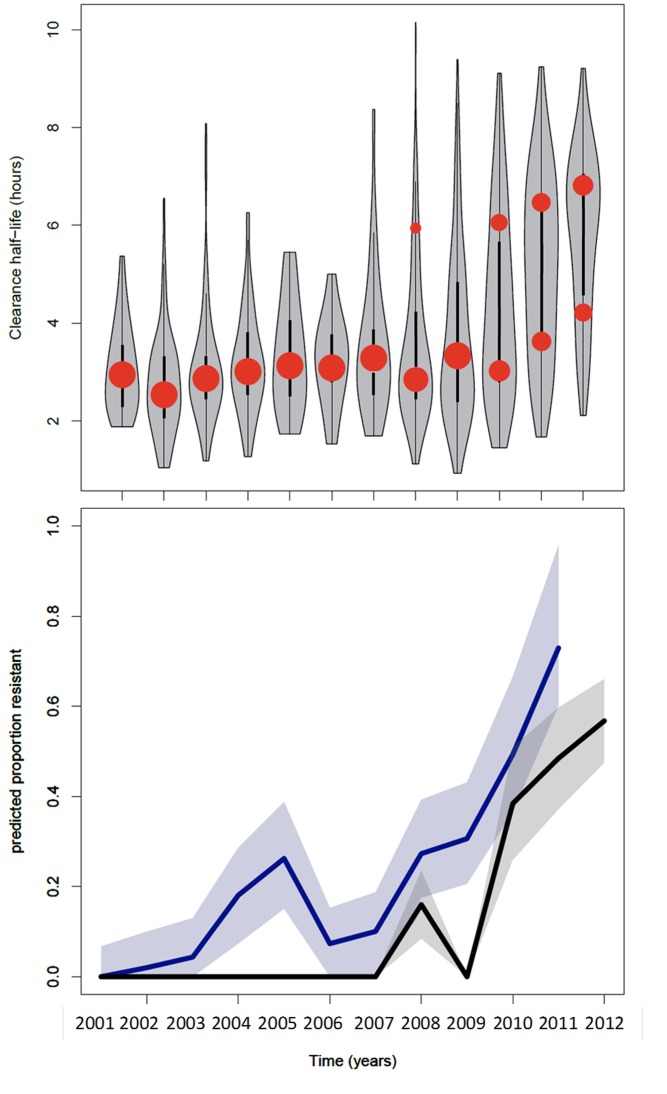
Summary of data from the Thai-Myanmar border and corresponding model results. **Top:** plot of the model fits (red dots) to observed data (grey violin plots, with a black bar representing the interquartile range) for the longitudinal study on parasite clearance after artemisinin treatment on the Thai-Myanmar border stratified by year. The number of dots per year indicates the number of contributing distributions estimated by the model. The position of the dot on the y-axis represents the geometric mean of the distribution, and the size of the dot represents the relative contribution of that subgroup to the full distribution. **Bottom:** plot of the predicted proportion of resistant infections (solid line) with 95% prediction interval (shaded area) using the mixture model (black) and the simulation model (blue).

The data from the Thai-Myanmar border, sorted by calendar year 2001–2012 are summarised in Table 1 and represented by violin plots in Fig 2.

**Table 1:** Summary of the data from the Thai-Myanmar border stratified by year.

	2001	2002	2003	2004	2005	2006	2007	2008	2009	2010	2011	2012
**N**	27	91	99	57	19	12	28	388	240	143	123	119
**Median**	2.8	2.6	2.8	3.0	3.2	3.2	3.1	3.1	3.3	4.0	5.3	6.2
**25** ^**th**^ **Percentile**	2.3	2.1	2.5	2.5	2.5	2.8	2.5	2.4	2.4	2.8	3.7	4.6
**75** ^**th**^ **Percentile**	3.5	3.3	3.3	3.8	4.1	3.8	3.9	4.2	4.8	5.7	6.6	7.0
**Source**	[18]	[18]	[18]	[18]	[18]	[18]	[18]	[18]	[18]	[18]	[18]	[18]

**Fig 3:**
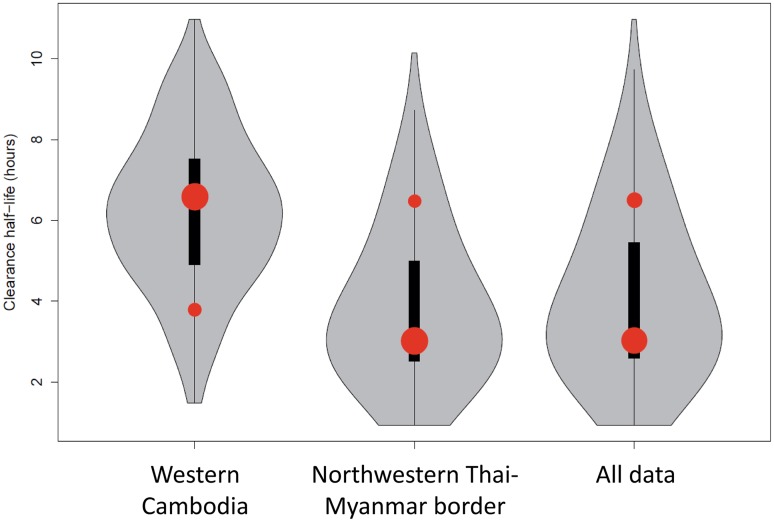
Plot of the model fits (red dots) to observed data (grey violin plots, with a black bar representing the interquartile range) aggregated by country. Number of dots per site indicates the number of contributing distributions. The position of the dot on the y-axis represents the geometric mean of the distribution and the size of the dot represents the relative contribution of that subgroup to the full distribution.

The full dataset was also disaggregated by country, summarised in Table 2 and represented by violin plots in Fig 3.

**Table 2:** Summary of the data stratified by country.

	All data	Northwestern Thai-Myanmar border	Western Cambodia
**N**	1,518	1,346	172
**Median**	3.5	3.3	6.2
**25** ^**th**^ **Percentile**	2.6	2.5	4.9
**75** ^**th**^ **Percentile**	5.5	5.0	7.5
**Source**	[16–18,20,21]	[18]	[16,17,20,21]

### Mixture Models

The most parsimonious model for the aggregate (observed) data from the three studies was a two-component model with each contributing component following a log-normal distribution (Fig 2 and Fig 3). For the distribution with the shortest half-life (henceforth referred to as the ‘sensitive’ distribution), the log_e_ of the half-life had a mean of 1.1 and a standard deviation of 0.37 corresponding to clearance half-lives with a geometric mean of 3.0 hours (95% CI 1.5–6.3; IQR 2.4–3.9). For the distribution with the longest half-life (henceforth referred to as the ‘resistant’ distribution), the log_e_ of the half-life had a mean of 1.9 and a standard deviation of 0.20 corresponding to clearance half-lives with a geometric mean of 6.5 hours (95% CI 4.4–9.6; IQR 5.7–7.4).

Allowing higher numbers of contributing components did not improve the overall fit of the model, indicating that if there were subgroups of resistant infections, this method combined with the data available was not able to identify them. This could be due to low sample sizes and/or the distributions of clearance half-lives of different resistant subgroups being similar as illustrated by the C580Y, R539T, and Y493H Kelch polymorphisms whose interquartile ranges of parasite half-lives closely overlap [7]. The ability of the model to differentiate between subpopulations depends on the means and standard deviations of the component populations as well as the sample size. A simulation experiment was performed to explore the limits of the model’s predictive power assuming the characteristics of the data analysed here. For a sample size of 50, the model was able to differentiate between subpopulations of geometric mean half-lives with a difference of 3 or more hours. The mixture model becomes more predictive with increasing sample size and is able to differentiate subpopulations whose geometric mean half-lives differ by only 0.5 hours for a sample size of 1,000. The results of this analysis are shown in detail in S1 Text. For a sample size of 1,000, the model identified input mixture distributions of 1, 2, 3, 4, and 5 components with an accuracy of 96%, 91%, 70%, 46%, and 21%, respectively, with the higher n-component distributions mostly being incorrectly predicted as (n-1)-component distributions. Details of this analysis can also be found in S1 Text and in the R code at https://github.com/lisawhite100/PDpheno-mixture.git.

The model was applied to parasite half-life data from the Thai-Myanmar border, sorted by calendar year 2001–2012, during which period artemisinin resistance emerged (Fig 2). The proportion of resistant infections was estimated from the best fit of the mixture model to the observed data. In cases where the model predicted a mixture of two components, the component with the higher geometric mean half-life described the proportion of resistant infections in that year. The first year in which the mixture model method identified a separate resistant population on the Thai-Myanmar border was in 2008. Histograms with the predicted composite distribution from the model fit are shown in Supporting Information file S2 Text. The data were also disaggregated by country to estimate the proportion of resisant infections in each country (Fig 3). The Akaike Information Criterion (AIC) values can be found in S2 Text.

### Defining the Phenotype

**Fig 4:**
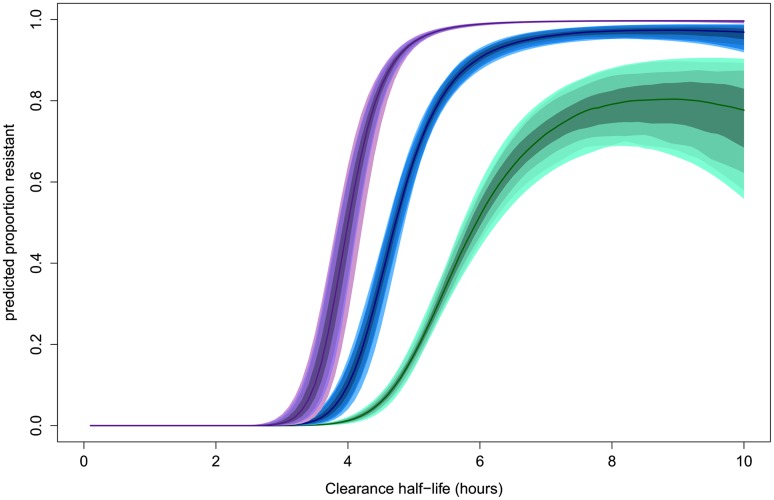
Model predictions for the probability that an infection with a given clearance half-life is resistant. This relationship is predicted to be dependent on the underlying proportion of ‘resistant’ infections in the study population. The relationships for underlying proportions ‘resistant’ of 0.1 (green), 0.5 (blue), and 0.9 (purple) are shown. The shaded areas represent the 50%, 80%, 90%, and 95% prediction intervals (from dark to light shading, respectively).

The estimated ‘sensitive’ and ‘resistant’ distributions of half-lives from the mixture model overlap. This is also the case for the Wild type and each of the C580Y, R539T, and Y493H polymorphisms [7]. The extent and position of the overlap depends on the size of each sub-distribution and thus its relative contribution to the full distribution. The overlap of ‘sensitive’ and ‘resistant’ distributions means that there is not a single cut-off value in parasite half-life to denote an infection as ‘sensitive’ or ‘resistant’. The mixture model can estimate the background proportion of ‘resistant’ infections without the requirement of assigning a ‘resistant’ or ‘sensitive’ label to each infection. This can then be used to assign a probability for an individual infection to belong to a ‘resistant’ or ‘sensitive’ parasite half-life distribution. This provides a much more accurate assessment than using a single half-life cut-off denoting resistance, since intermediate clearance half-lives can be part of the lower tail end of the ‘resistant’ parasite half-life distribution or the top-end of the ‘sensitive’ one. The mixture model predicts that the relationship between the likelihood that an individual infection is ‘resistant’ and the observed parasite clearance half-life is for the individual patient not the same in every setting. This relationship is predicted to change depending on the underlying proportion of ‘resistant’ infections from which the individual is sampled (Fig 4). This effect is demonstrated by choosing three underlying proportions of the population to be ‘resistant’ (0.1, 0.5, and 0.9). For each of the three example populations (with low, medium, and high levels of resistance), the fitted model is then used to predict the proportion of the mixture distributions representing the ‘resistant’ component of the mixture for each of a range of half-lives. The probability of an individual with a given half-life belonging to the ‘resistant’ component would therefore be predicted by this proportion. Fig 4 shows this relationship using the mixture model (see https://github.com/lisawhite100/PDpheno-mixture.git for the full R code and demonstration datasets). Using this approach, the average cut-off in parasite half-life denoting resistance could be any time between 3.5 and 5.5 hours (the values of the half-life where the expected proportion of resistant infections is 0.5, Fig 4), depending on the underlying proportion of resistant infections. Fig 4 also shows that it is not possible to predict on an individual level with certainty from a single parasite half-life that an infection is resistant.

### Comparison with Distributions Defined by Genetic Relatedness

We explored whether the contributing distributions derived by our algorithm describing the observed data remained accurate when independent measures of genetic relatedness (‘clonality’) between parasite strains were taken into consideration. This was indeed the case: from 100 datasets where the clone number was randomly assigned, the most parsimonious log-normal mixture model was unimodal in all but four cases.

### Application to Routine Surveillance

**Fig 5:**
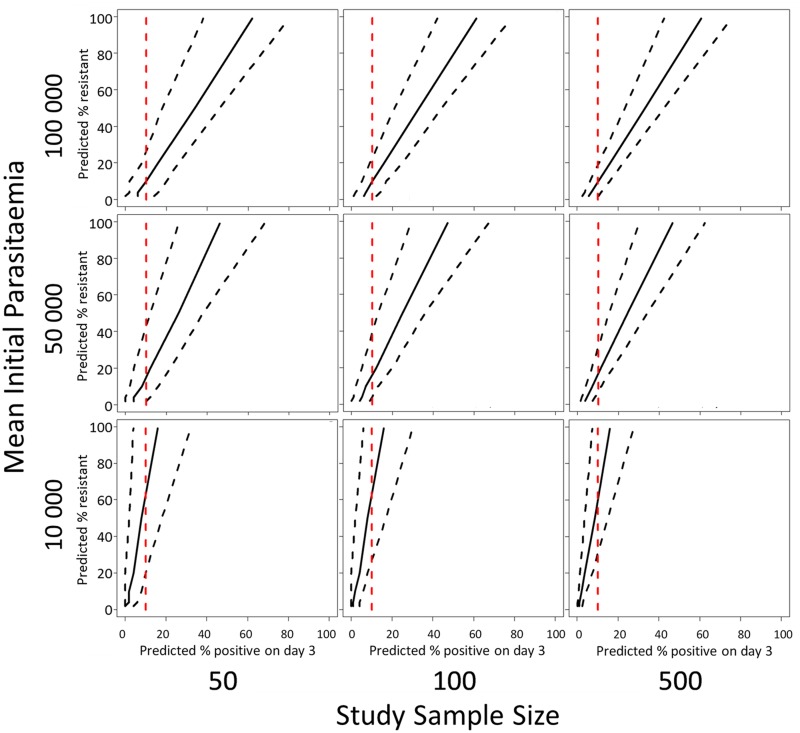
A plot of the simulation model prediction for the relationship between the percentage of patients parasitaemic after 3 days of treatment and the percentage of ‘resistant’ infections in the sample (solid black lines) with 95% prediction interval (dashed black lines). This prediction is plotted with a 10% threshold for the percentage of patients positive on day-3 of treatment (red dashed line). The rows of the panel represent three different assumptions about the geometric mean parasitaemia on admission and the columns of the panel represent three different assumptions about the study sample size.

The model predictions of the relationship (with 95% prediction intervals) between the proportion of patients with parasites on the third day of treatment and the proportion of ‘resistant’ infections in a population defined by parasite clearance half-life are illustrated in Fig 5. Since the proportion of patients still carrying parasites on the third day will depend on the initial parasitaemia, and the prediction precision will depend on the sample size, results are given for three different assumptions of geometric mean parasitaemia on admission (10,000, 50,000, and 100,000) and three different assumptions about the study sample size (50, 100, 500). Fig 5 shows the range in the estimated proportion of ‘resistant’ infection based on the currently recommended 10% threshold for parasitaemia three days after drug treatment [25]. The row showing results for a mean initial parasitaemia of 50,000 corresponds with the two datasets analysed in this study with the first column corresponding to the annual sample sizes, and the second and third columns corresponding to the sample sizes of aggregated data [20].

For the intermediate admission parasitaemias (50,000/μL, Fig 5 middle row), an observation of 10% of patients with parasites on ‘day-3’ is consistent with a model prediction of a proportion of resistant infections being statistically different from zero. For high admission parasitaemias (>50,000/μL, Fig 5 top row) and small sample sizes [50], the model results indicate that a higher threshold like 20% would be more appropriate. For a lower geometric mean admission parasitaemia (<50,000/μL, Fig 5 bottom row), the model results indicate that a lower threshold of 5% would be more appropriate (see Supporting Information file S3 Text for full results of simulations for days 1, 2, and 3).

The model was used to simulate the proportions of patients with parasites on days 1, 2, and 3 of treatment between 2001 and 2011 using data from the study on the northwestern border of Thailand in patients with uncomplicated hyperparasitaemic (>4%) *falciparum* malaria treated with various oral artesunate-containing therapies [18]. The most likely estimates for the proportion ‘resistant’ over time using this method are plotted in Fig 2 (bottom graph, blue). When compared with the mixture model prediction (Fig 2, bottom graph, black), the simulation model (Fig 2, bottom graph, blue) predicts the presence of resistance earlier (significantly different from zero in 2004–5 and 2008–12) and at higher levels. This is because the mixture model approach uses a stricter definition of resistance than the simulation model approach. The comparison of the outputs of the two approaches highlights the issue that predictions about the presence and relative frequency of resistance in a population are dependent on the underlying assumptions about the ‘sensitive’ and ‘resistant’ subgroups and the choice of significance level for the selection of competing models.

## Discussion

We propose a mathematical approach to predict the distributions of clearance half-lives for artemisinin-sensitive and-resistant infections after artemisinin treatment. This approach uses only pharmacodynamic phenotypic data. The observed data were best represented by two groups with different clearance half-lives, assumed to represent artemisinin-resistant and-sensitive infections. Based on this result, we propose a sensitive population with a geometric mean parasite half-life of 3 hours and a resistant population with a geometric mean half-life of 6.5 hours. The findings were supported by the fact that the model estimates did not change when the model was applied to the half-life estimates for the geometric mean half-lives of multiple infections with the same clones. The bimodal distribution in half-lives does not imply that the development of artemisinin resistance is a one-step process, although the recent discovery that a single mutation in the propeller region of the Kelch protein is strongly associated with artemisinin resistance [7] and a very similar distribution of clearance half-lives as defined by the described model are consistent with a one-step process. Using the dataset from the Thai-Myanmar border stratified by year [18], the most parsimonious models for the early years were single distributions whose means did not increase with time, followed by double distributions in later years. This pattern provides evidence of the emergence of a distinct subgroup rather than a gradual shift in half-life. For the C580Y, R539T, and Y493H Kelch polymorphisms, distributions of half-lives described in [7] are (mean, IQR) 7.19 hours (6.47–8.31), 6.64 hours (6.00–6.72), and 6.28 hours (5.37–7.14), respectively. Each distribution is similar to the others and to the predicted ‘resistant’ distribution from the mixture model with clearance half-lives with a geometric mean of 6.5 hours (95% CI 4.4–9.6; IQR 5.7–7.4).

A limitation of this approach is the assumption that the clearance half-lives of infections with sensitive or resistant parasites follow unimodal distributions. Although a number of distribution types were explored, with the log-normal distribution being the most likely, there is no guarantee that this assumption is justified. Moreover, distributions which are too similar (like those of the C580Y, R539T, and Y493H polymorphisms) would not be separately identified with this method. The accuracy of the approach also reduces with increasing numbers of components and reducing sample size.

Generalisation of the study findings to a high-transmission setting, such as regions of Sub-Saharan Africa, will need additional evaluation. Parasite clearance is accelerated in semi-immune individuals in high-transmission settings, and high prevalence of mixed or multi-clonal infections in such settings could also affect clearance characteristics. Multi-clonal infections were excluded from the current study. A direct application of the mixture model approach is that in studies describing parasite clearance rates and half-lives, it could be used to assess the likelihood that observations of prolonged clearance are either the upper tail end of a log-normal distribution or denote a separate population of artemisinin-resistant parasites. In the longitudinal study from the Thai-Myanmar border, it thus becomes clear that as early as 2008 a separate parasite population with the slow clearance phenotype could be identified. This would not be apparent from the change in median or mean half-life of all parasites in that year compared with other years. The approach also provides a clear estimate of the proportion of resistant infections over time in the same area, which is a much more accurate representation than reporting the proportion of patients with a parasite half-life above an ill-defined cut-off value. As shown in our analysis, this is because such a cut-off value will depend on the size of the contributing underlying proportion of resistant and sensitive parasites. An interesting observation from the longitudinal data from the Thai-Myanmar border was the increase in geometric mean half-life in both the sensitive and resistant populations in 2012. A possible explanation is increased resistance to the partner drug mefloquine, which emerged more prominently during that period.

The approach described in this study also provides a method to select parasite strains with a defined probability of being artemisinin-resistant according to their *in vivo* parasite half-life. This provides an essential tool for the interpretation of genome-wide association studies and studies for further validation of the recently identified molecular markers and population structures strongly associated with artemisinin resistance [5,7]. The importance of an adequate *in vivo* phenotypic definition has recently been emphasized again, since interpretation of newly discovered Kelch 13 mutations without phenotypic information has proven cumbersome. In addition, novel *in vitro* susceptibility tests for artemisinin derivatives focussing on the resistance of ring stage parasites can be calibrated against the *in vivo* phenotype in a similar way.

Finally, our study also provides a rationale for the choice of a cut-off level denoting artemisinin resistance for the proportion of patients still parasitaemic after 3 days of antimalarial treatment containing artemisinins. The current WHO recommended cut-off level of 10% [25] as a warning sign for suspecting artemisinin resistance is acceptable in several scenarios, but is heavily dependent on the initial parasitaemia at the start of the ACT treatment. It shows that reports providing proportions of patients still parasitaemic at ‘day-3’ should include the distribution of parasite density on initiation of therapy. It is also apparent that the confidence interval of the level of resistance related to ‘day-3’ positiveness is wide and dependant on the size of the population evaluated. In some cases 10% of patients still parasitaemic on the third day can include the absence of artemisinin resistance (for example, if the initial parasitaemia was very high or the sample size was low), and in other cases the 10% threshold may indicate a large proportion of resistant infections (for example, when the admission parasitaemia is low).

## Conclusion

Our study shows that analysing data on parasite half-life as distributions of artemisinin-sensitive and artemisinin-resistant populations each with distinct geometric means is valid and has important advantages for the interpretation of study results. It provides a tool for early recognition of a resistant parasite population in a geographical area. It also quantitates the probability that a parasite strain with a given parasite half-life is resistant to artemisinins, which is important for the evaluation of novel artemisinin *in vitro* susceptibility tests and molecular markers. The WHO—recommended definition of suspected artemisinin resistance in cases where more than 10% of patients are still parasitaemic on the third day of treatment is useful, but should be interpreted by including the initial parasitaemia. The ‘day-3’ cut-off lacks accuracy in predicting the real proportion of artemisinin-resistant parasites, and should thus be followed by a more detailed assessment.

## Supporting Information

S1 Text Simulation approach to assess the choice of model selection criterion. (DOCX)

S2 Text Data plotted with predicted composite distributions from mixture models. (DOCX)

S3 Text Simulation of day 1, 2, and 3 percentage positive observations. (DOCX)

## Abbreviations:

ACT: Artemisinin combination therapy
SNPs: single nucleotide polymorphisms
