# Supplementary material for: Defining the In Vivo Phenotype of Artemisinin-Resistant Falciparum Malaria: A Modelling Approach
Source: PLoS Med. 2015 Apr 28;12(4):e1001823. doi: 10.1371/journal.pmed.1001823 (PMC4412633; doi:10.1371/journal.pmed.1001823)
Supplement: S1 Text — (DOCX) [file pmed.1001823.s001.docx]

**Defining the *in-vivo* phenotype of artemisinin resistant *falciparum* malaria: A modelling approach**

**Supporting information 1 – Simulation approach to assess the choice of model selection criterion**

The ability of the model to differentiate between subpopulations depends on the means and standard deviations of the component populations as well as the sample size. A simulation experiment was performed to explore the limits of the models predictive power assuming the characteristics of the data. A series of samples from composite 2-component log-normal distributions where the first “sensitive” component distribution had a log_e_ mean half-life of 1.1 and a standard deviation of 0.37 corresponding to clearance half-lives with a geometric mean of 3.0 hours (95% CI 1.5–6.3; IQR 2.4-3.9). The second “resistant” component had the following range of characteristics:

- geometric mean of 3.5 hours and a standard deviation (based on log_e_ half-lives) of 0.20
- geometric mean of 4.0 hours and a standard deviation (based on log_e_ half-lives) of 0.20
- geometric mean of 4.5 hours and a standard deviation (based on log_e_ half-lives) of 0.20
- geometric mean of 5.0 hours and a standard deviation (based on log_e_ half-lives) of 0.20
- geometric mean of 5.5 hours and a standard deviation (based on log_e_ half-lives) of 0.20
- geometric mean of 6.0 hours and a standard deviation (based on log_e_ half-lives) of 0.20
- geometric mean of 6.5 hours and a standard deviation (based on log_e_ half-lives) of 0.20

For each pair of “sensitive” and “resistant” components were mixed with following proportion “resistant”: 0.1, 0.2, 0.3, 0.4, 0.5, 0.6, 0.7, 0.8, 0.9. This process yielded to 63 simulated datasets. The generation of these 63 datasets was repeated assuming sample sizes of 50, 100, 200 and 1000.

The panel of graphs represents the ability of the model selection approach as applied to the true dataset to identify the components of the simulated composite distributions. For a sample size of 50, the model was able to differentiate between subpopulations of geometric mean half-lives with a difference of 3 or more hours. The approach becomes more predictive with increasing sample size and is able to differentiate subpopulations whose geometric mean half-lives differ by only 0.5 hours for a sample size of 1000.

Since the model was applied to aggregate data with a sample size of 1518, this simulation approach supports the ability of the selection criterion to identify two components correctly for a broad range of potential mean resistant half-lives. The disaggregated datasets ranged from sample sizes of 12 to 388, thus indicating that for the expected difference in geometric mean derived from analysis of the full dataset, the selection criterion would in most cases identify two components correctly should they exist.

In order to assess the ability of the approach to correctly identify the number of components of a mixture distribution, a further experiment was performed. 1000 mixture distributions were created comprising each of 1, 2, 3, 4, 5 components. Each component followed a log-normal distribution. The sample size for each composite distribution was 1000. The mean of each component distribution was sampled from the ranges: 0 to 2, 2 to 4, 4 to 6, 6 to 8 and 8 to 10 without replacement to avoid overlapping component distributions. The relative proportion of each component was randomly assigned. The standard deviation of each component was randomly assigned from the range 0 to 0.2. The model and parsimonious selection criterion was applied to each input composite distribution and the predicted number of components was compared with the input number of components. Below is a plot of the predicted number of components compared with the input number. The solid line represents the median of the predicted number of components; the dashed line represents the mean of the predicted number of components, the dark grey shaded area represents the interquartile range and the light grey shaded area represents the 5 to 95 percentile range.

Below is a plot of the percentage of model fits which correctly predicted the number of components.
