# Supplementary material for: Defining the In Vivo Phenotype of Artemisinin-Resistant Falciparum Malaria: A Modelling Approach
Source: PLoS Med. 2015 Apr 28;12(4):e1001823. doi: 10.1371/journal.pmed.1001823 (PMC4412633; doi:10.1371/journal.pmed.1001823)
Supplement: S2 Text — (DOCX) [file pmed.1001823.s002.docx]

**Defining the *in-vivo* phenotype of artemisinin resistant *falciparum* malaria: A modelling approach**

**Supporting information 2 - data plotted with predicted composite distributions from mixture models**


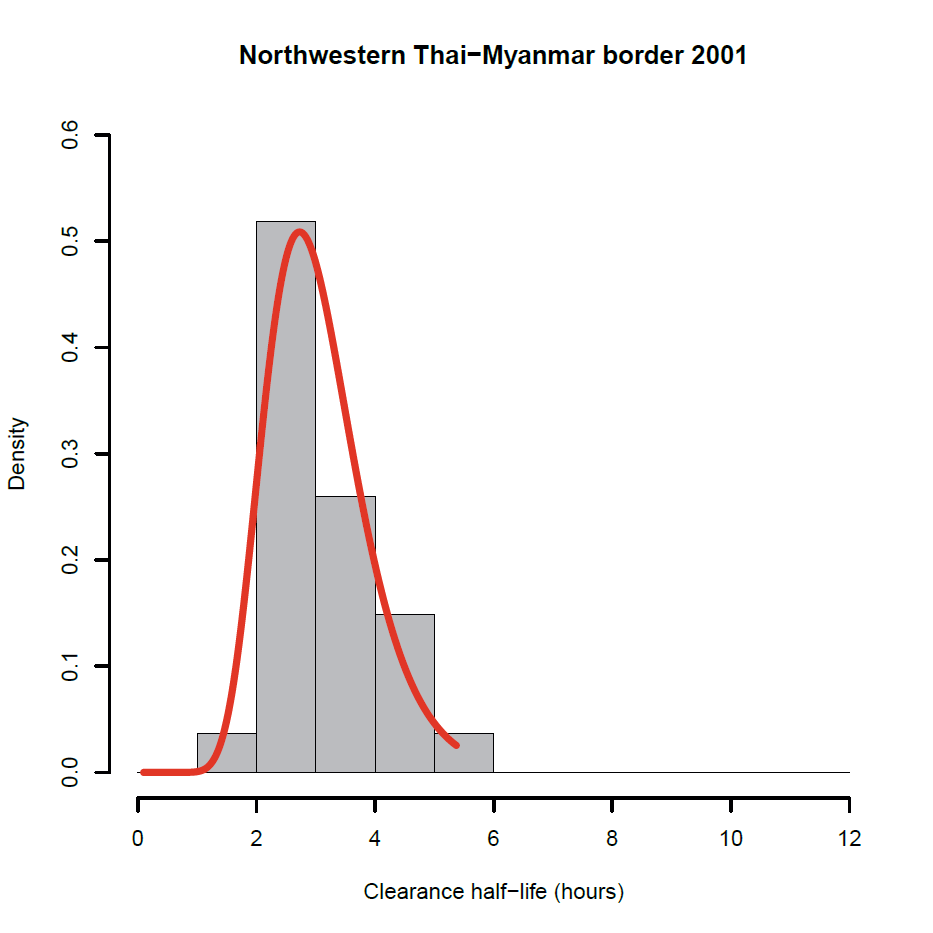


A plot of the most parsimonious model (red) as a probability density function (red line) with a histogram of the clearance half-life data (grey) for the Northwestern Thai-Myanmar border in 2001.


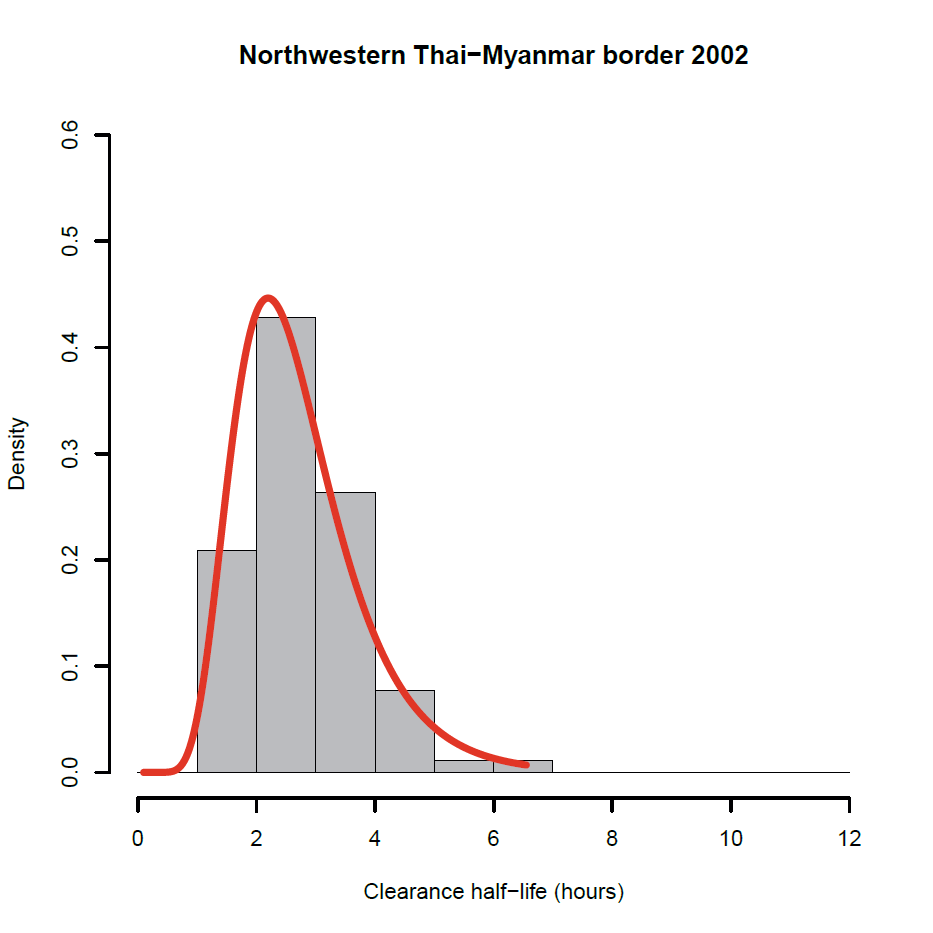


A plot of the most parsimonious model (red) as a probability density function (red line) with a histogram of the clearance half-life data (grey) for Northwestern Thai-Myanmar border in 2002.


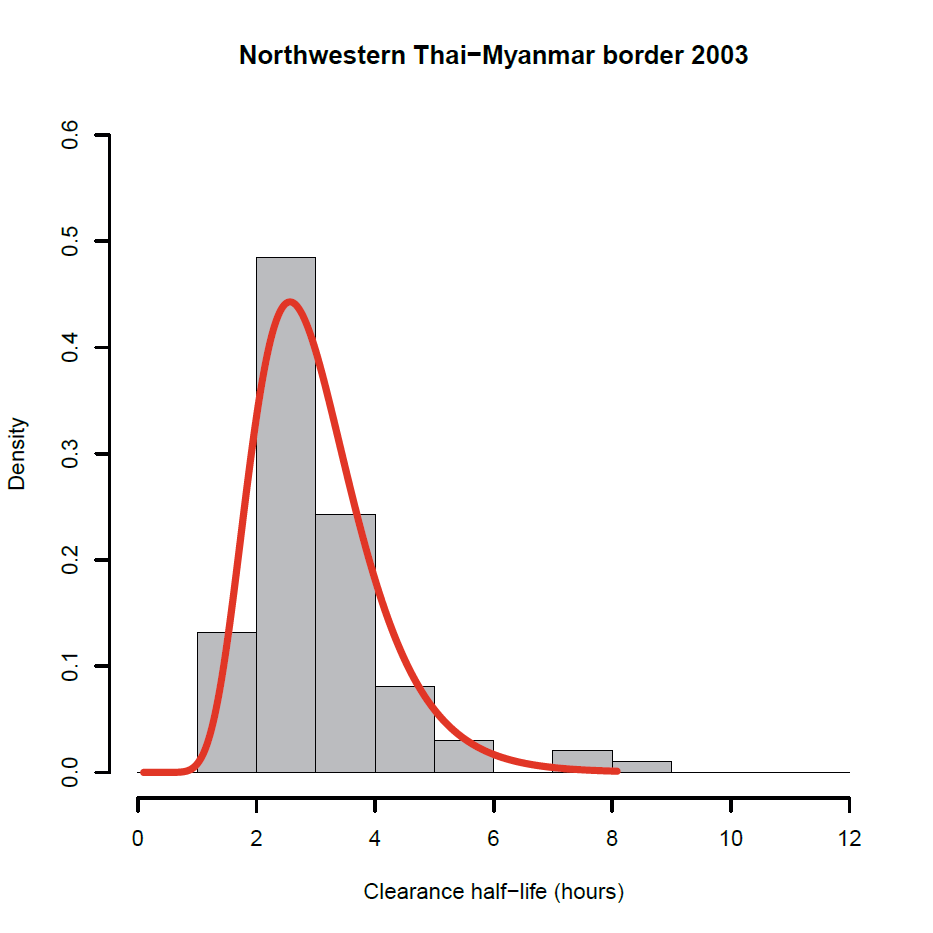


A plot of the most parsimonious model (red) as a probability density function (red line) with a histogram of the clearance half-life data (grey) for the Northwestern Thai-Myanmar border in 2003.


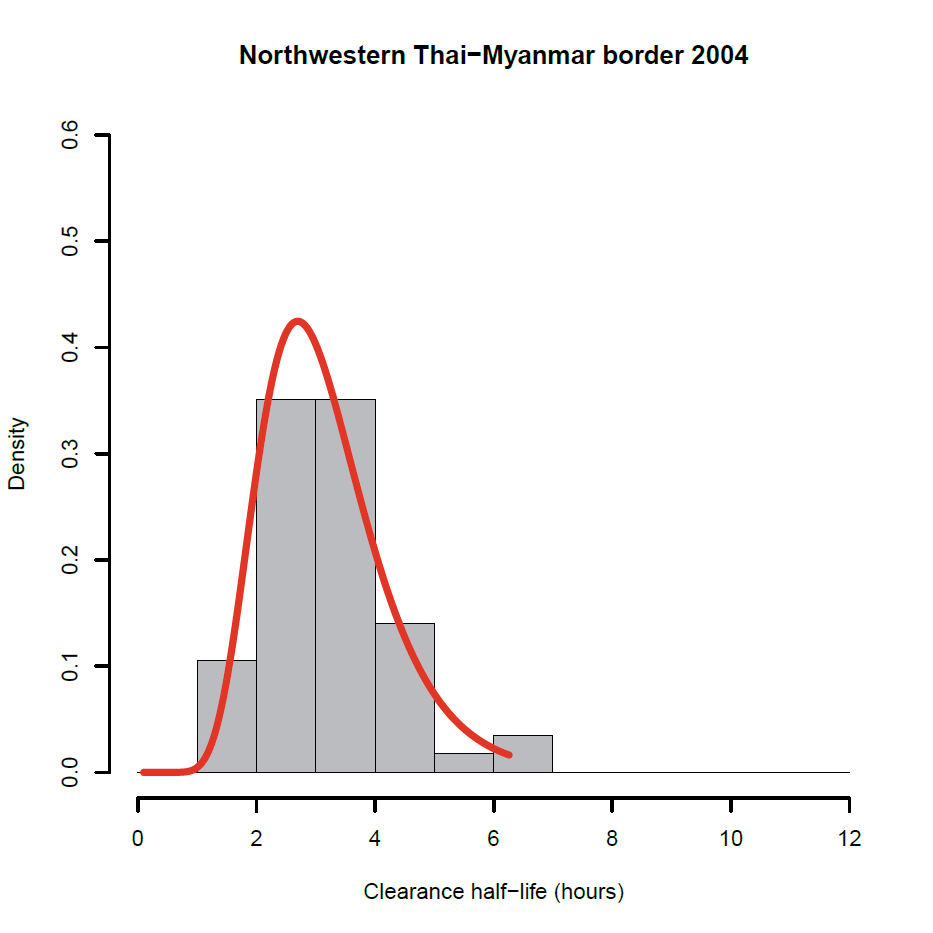


A plot of the most parsimonious model (red) as a probability density function (red line) with a histogram of the clearance half-life data (grey) for the Northwestern Thai-Myanmar border in 2004.


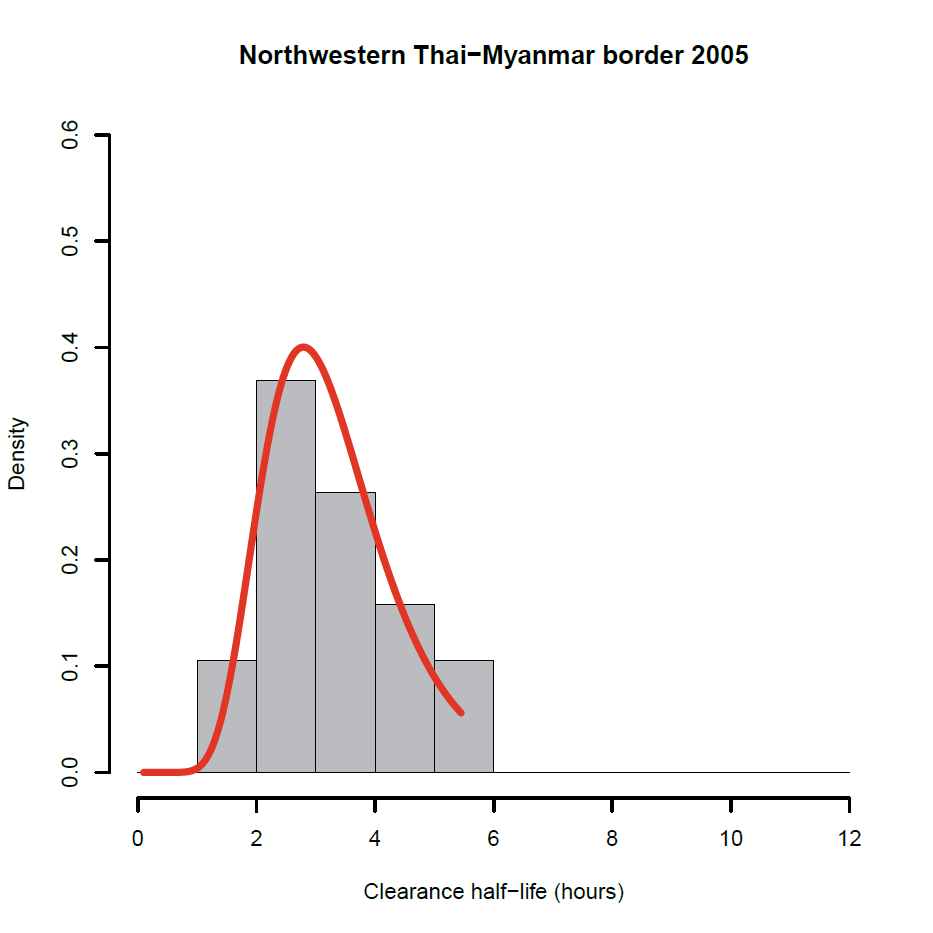


A plot of the most parsimonious model (red) as a probability density function (red line) with a histogram of the clearance half-life data (grey) for the Northwestern Thai-Myanmar border in 2005.


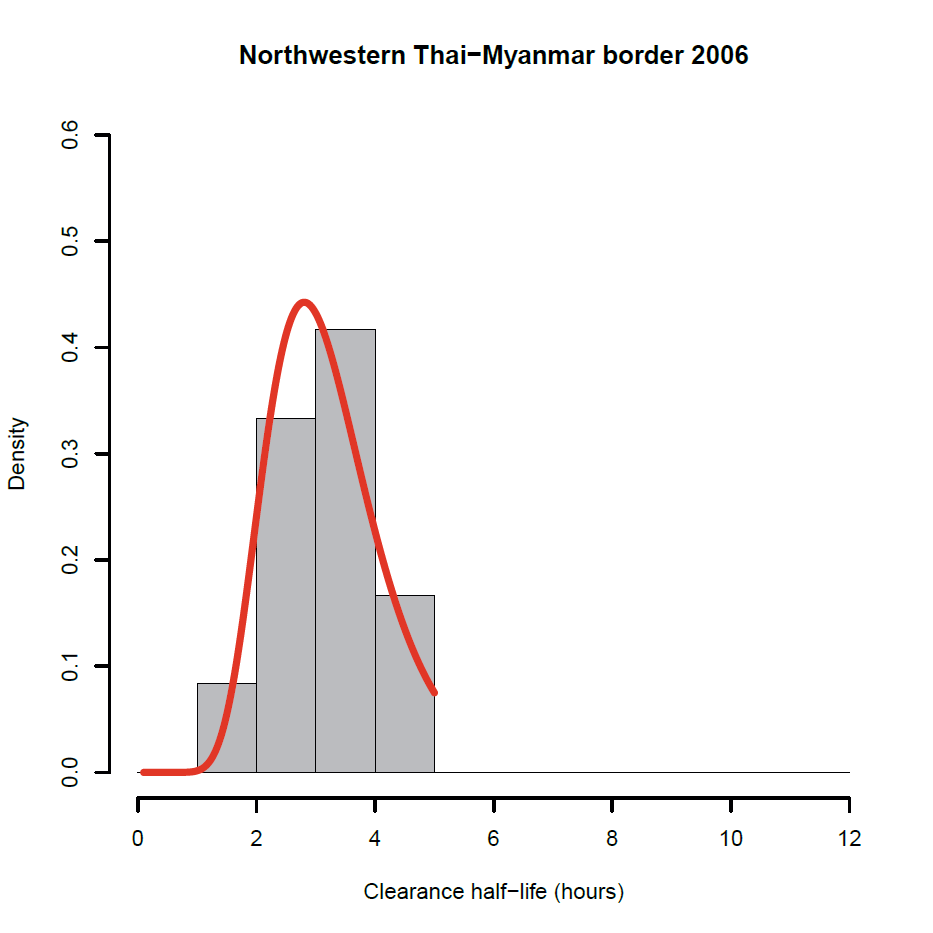


A plot of the most parsimonious model (red) as a probability density function (red line) with a histogram of the clearance half-life data (grey) for the Northwestern Thai-Myanmar border in 2006.


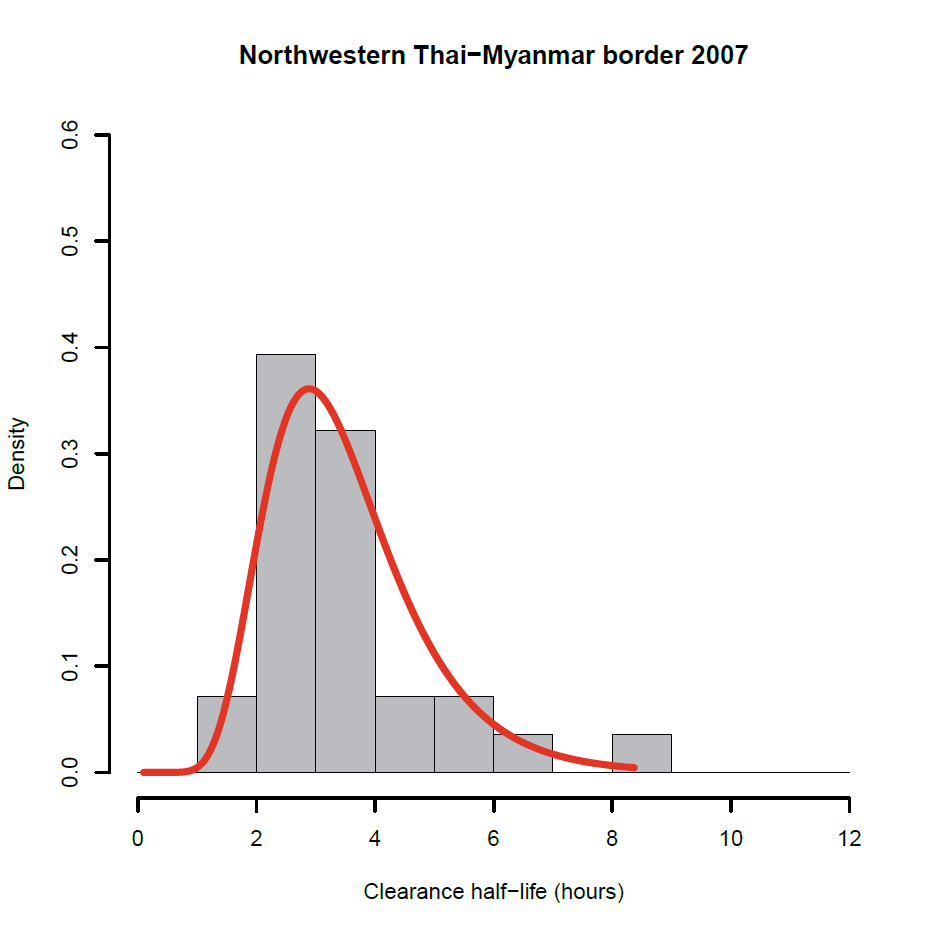


A plot of the most parsimonious model (red) as a probability density function (red line) with a histogram of the clearance half-life data (grey) for the Northwestern Thai-Myanmar border in 2007.


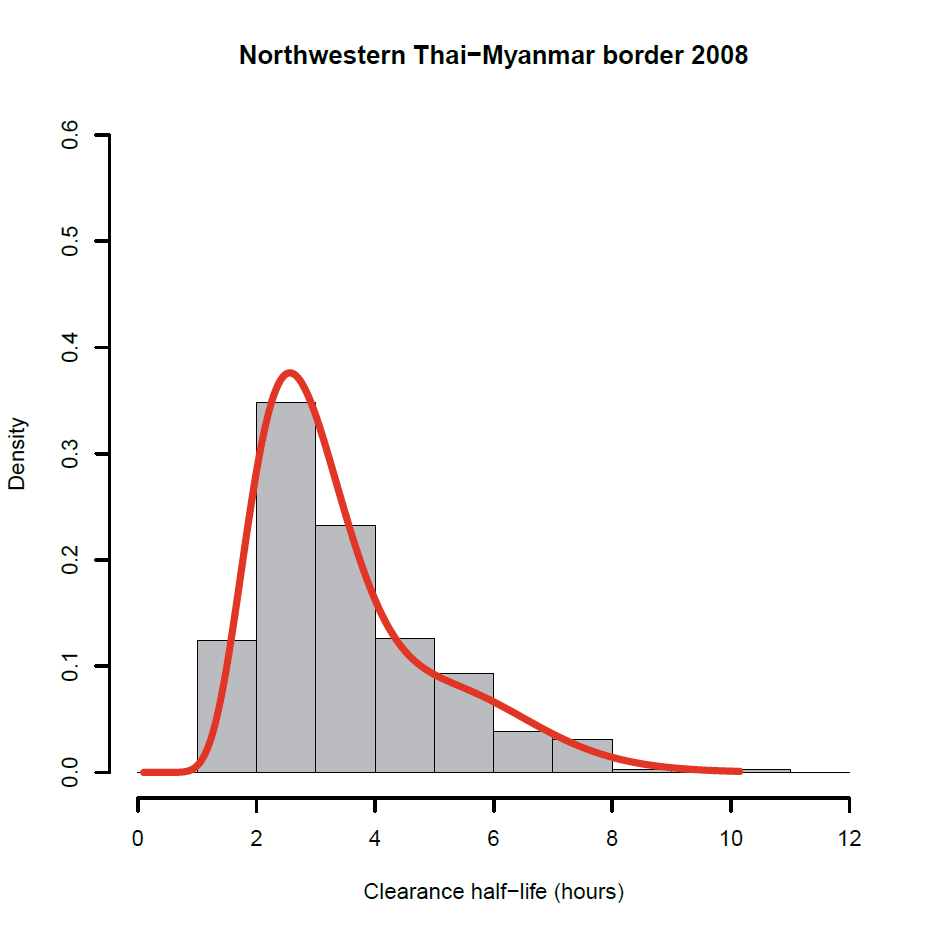


A plot of the most parsimonious model (red) as a probability density function (red line) with a histogram of the clearance half-life data (grey) for the Northwestern Thai-Myanmar border in 2008.


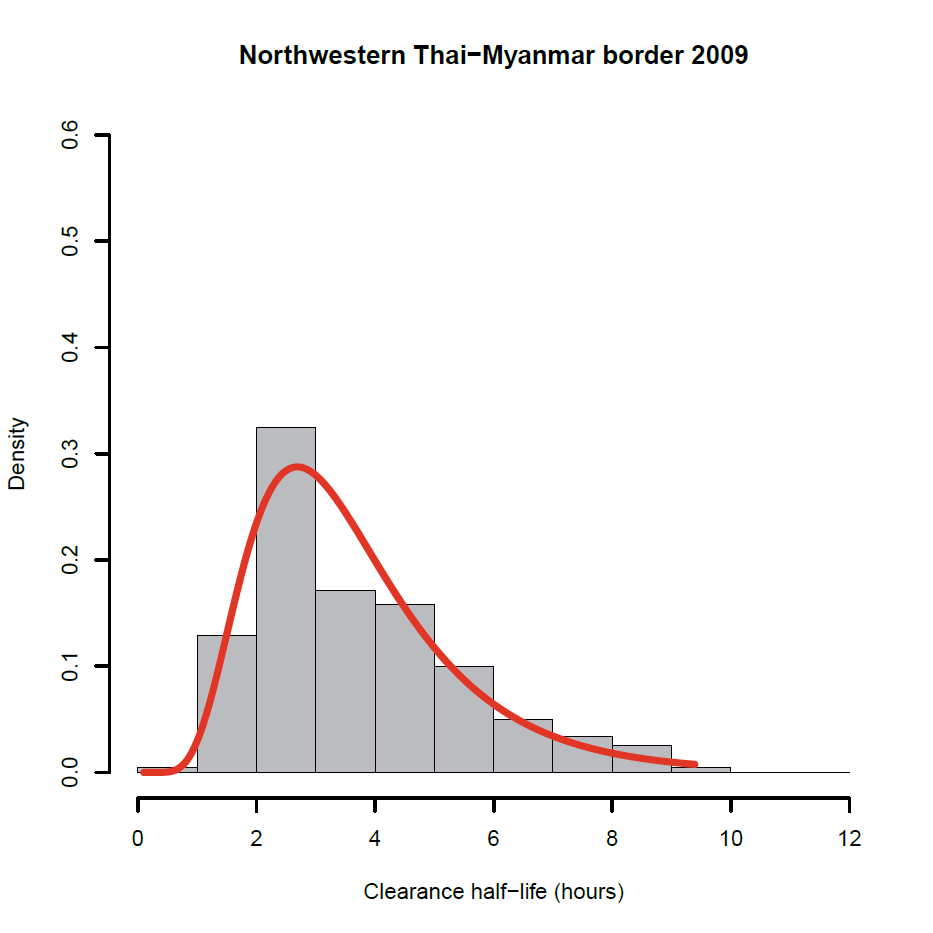


A plot of the most parsimonious model (red) as a probability density function (red line) with a histogram of the clearance half-life data (grey) for the Northwestern Thai-Myanmar border in 2009.


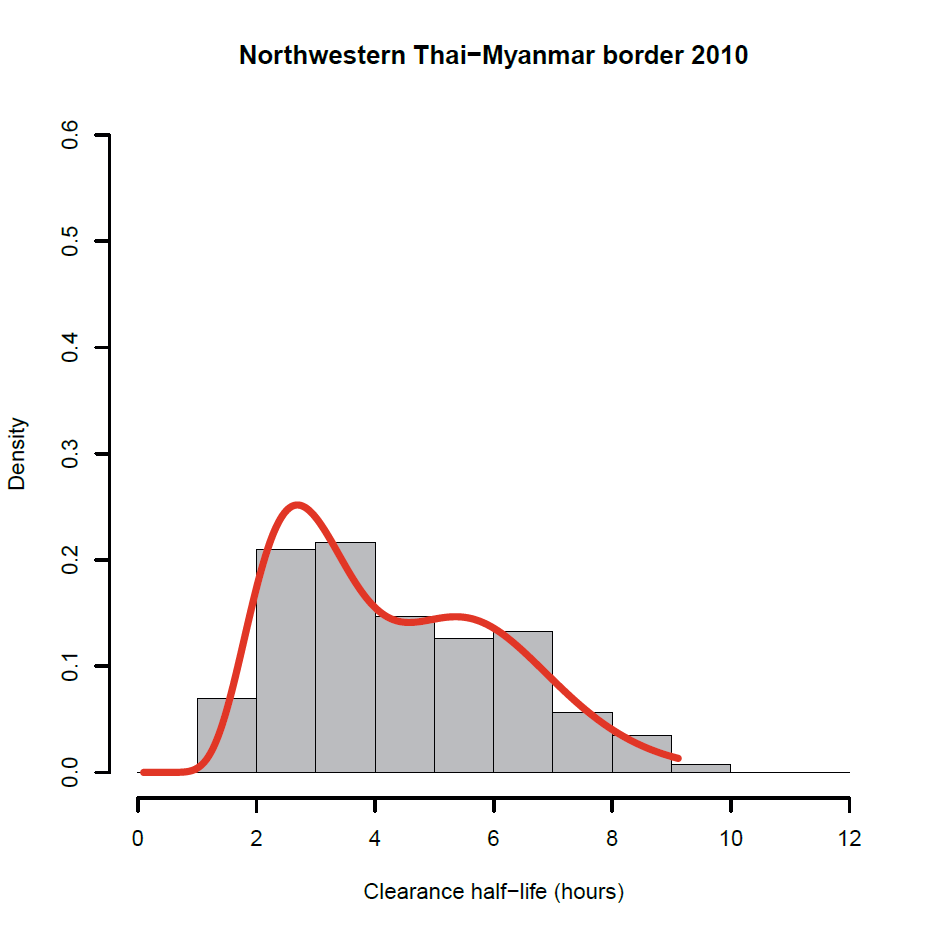


A plot of the most parsimonious model (red) as a probability density function (red line) with a histogram of the clearance half-life data (grey) for the Northwestern Thai-Myanmar border in 2010.


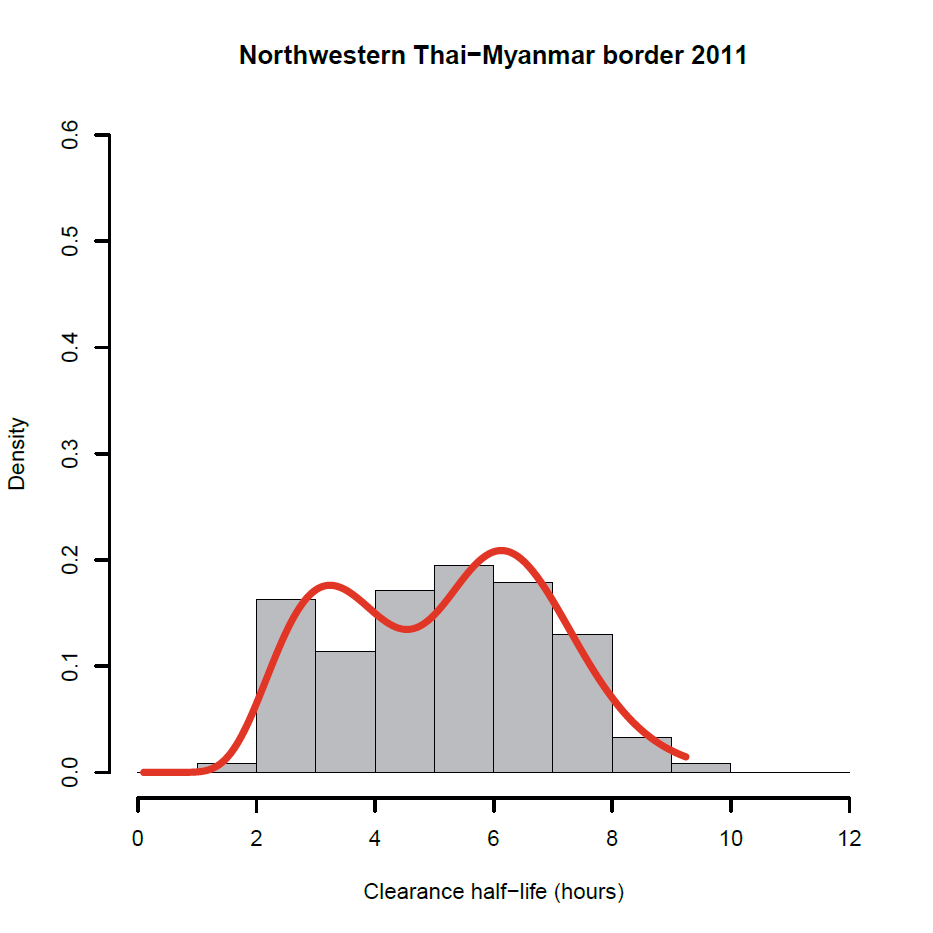


A plot of the most parsimonious model (red) as a probability density function (red line) with a histogram of the clearance half-life data (grey) for the Northwestern Thai-Myanmar border in 2011.


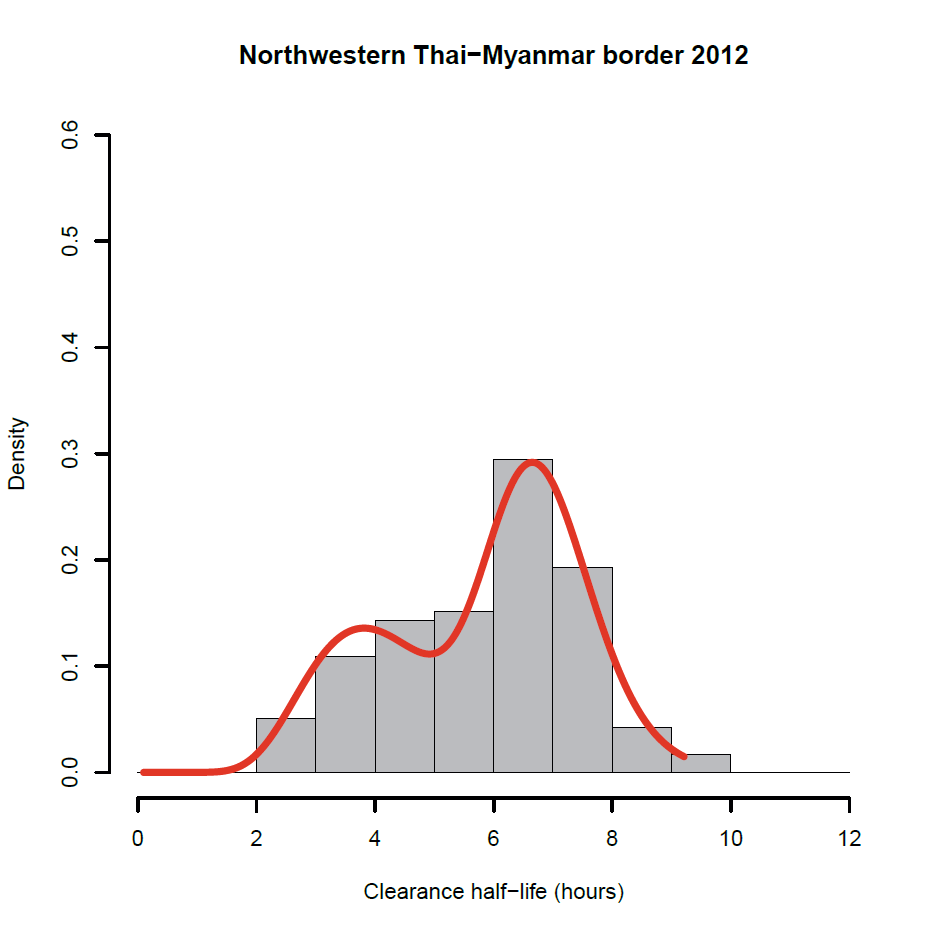


A plot of the most parsimonious model (red) as a probability density function (red line) with a histogram of the clearance half-life data (grey) for the Northwestern Thai-Myanmar border in 2012.

**AIC values for the North-Western Thai-Myanmar border stratified by year**

| Components | 2001 | 2002 | 2003 | 2004 | 2005 | 2006 | 2007 | 2008 | 2009 | 2010 | 2011 | 2012 |
| --- | --- | --- | --- | --- | --- | --- | --- | --- | --- | --- | --- | --- |
| 1 | 11.3 | 85.6 | 66.8 | 39.4 | 16.6 | 9.7 | 26.1 | 416.4 | 316.1 | 182.9 | 125.0 | 78.3 |
| 2 | 12.6 | 82.3 | 62.3 | 37.4 | 21.1 | 13.3 | 30.3 | 407.0 | 314.4 | 175.8 | 107.7 | 44.5 |
| 3 | 18.0 | 89.5 | 61.0 | 42.5 | 22.4 | 15.9 | 34.5 | 411.5 | 319.1 | 179.2 | 110.8 | 48.5 |
| 4 | 17.5 | 86.0 | 63.6 | 39.5 | 24.0 | 20.7 | 34.8 | 416.0 | 319.5 | 175.2 | 108.2 | 44.5 |
| 5 | 17.6 | 89.3 | 62.7 | 43.8 | 27.0 | 0.0 | 40.3 | 422.0 | 324.5 | 179.7 | 113.6 | 44.7 |

If the condition of minimum AIC is used rather than the condition for most parsimonious model given in the methods section of the main text, then the plot of the means of the best fitting mixture models compared with the violin plots of the data would be as follows:


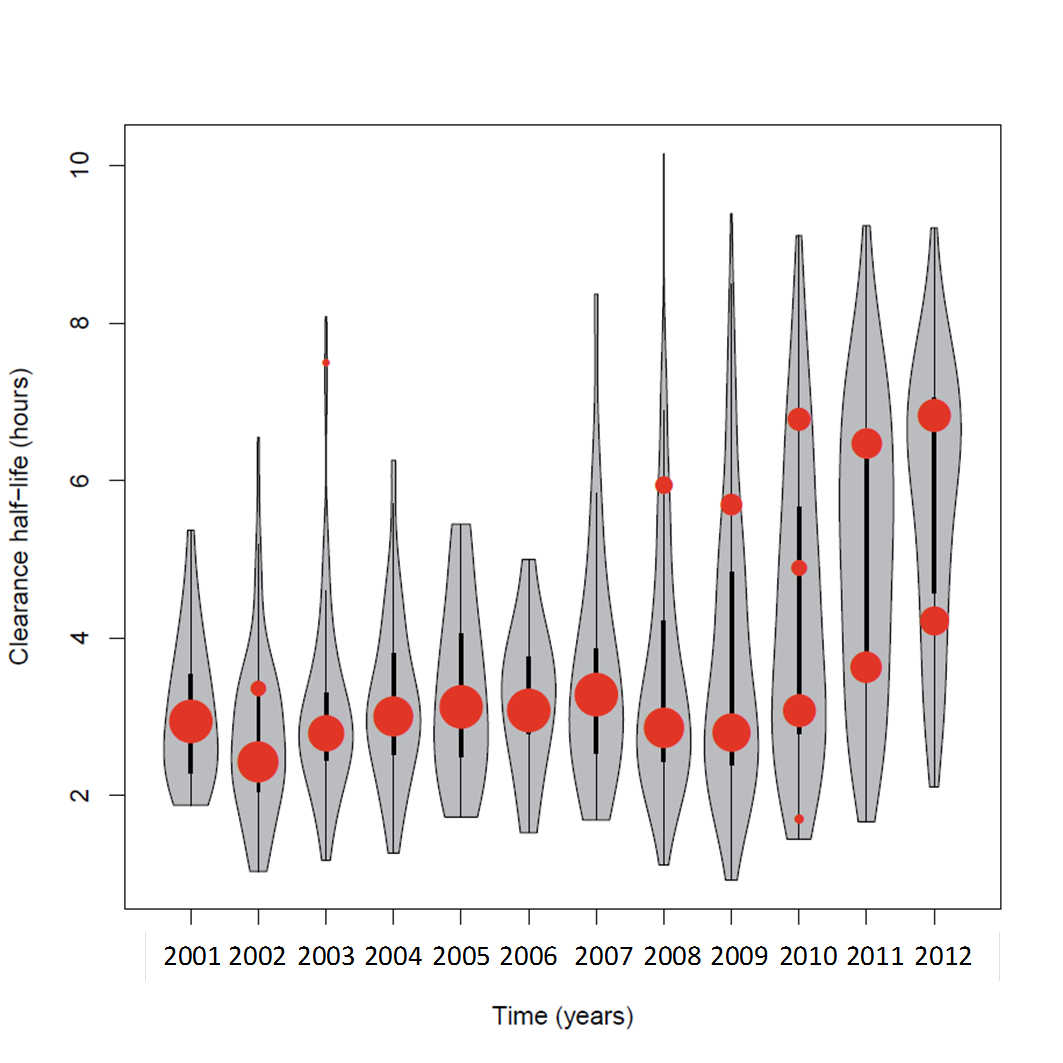


**AIC values for the full dataset stratified by country**

| Components | All data | Northwestern Thai-Myanmar border | Western Cambodia |
| --- | --- | --- | --- |
| 1 | 2043.9 | 1697.1 | 139.5 |
| 2 | 1928.3 | 1608.1 | 116.0 |
| 3 | 1932.1 | 1612.4 | 112.7 |
| 4 | 1927.9 | 1605.3 | 111.5 |
| 5 | 1933.3 | 1607.8 | 112.3 |

If the condition of minimum AIC is used rather than the condition for most parsimonious model given in the methods section of the main text, then the plot of the means of the best fitting mixture models compared with the violin plots of the data would be as follows:

**
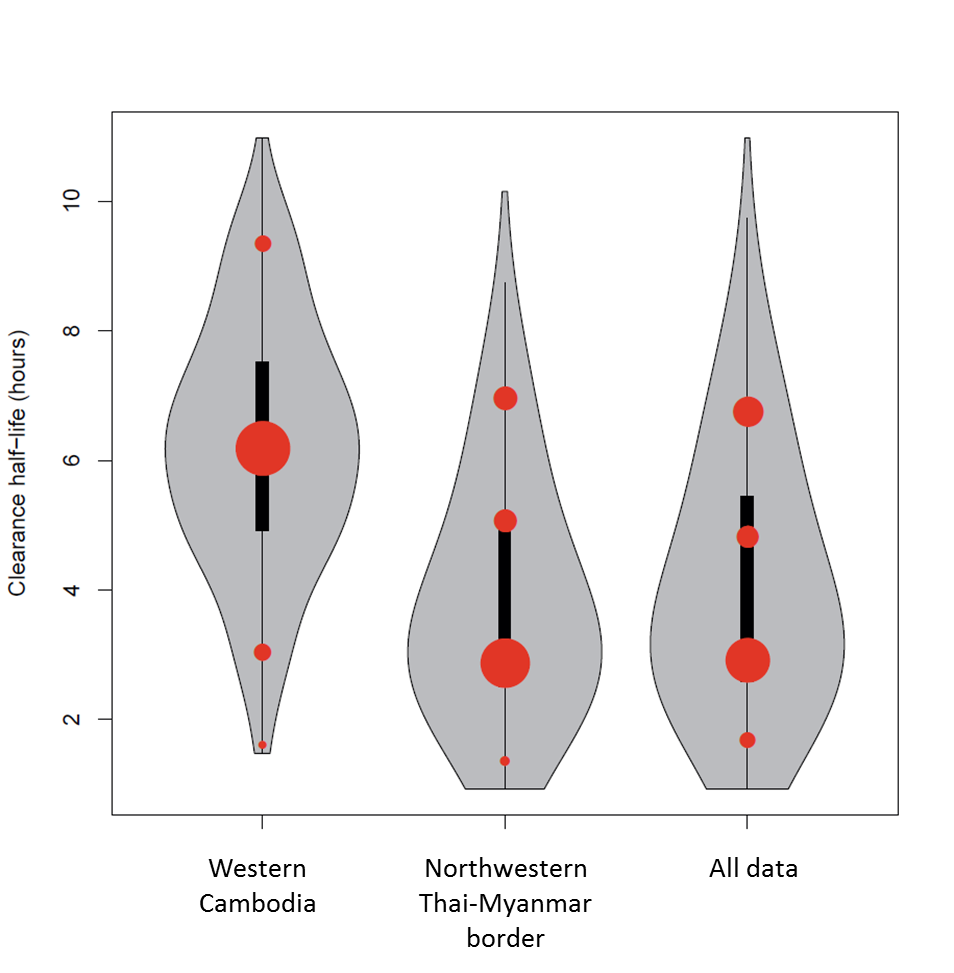
**
