# Supplementary material for: Defining the In Vivo Phenotype of Artemisinin-Resistant Falciparum Malaria: A Modelling Approach
Source: PLoS Med. 2015 Apr 28;12(4):e1001823. doi: 10.1371/journal.pmed.1001823 (PMC4412633; doi:10.1371/journal.pmed.1001823)
Supplement: S3 Text — (DOCX) [file pmed.1001823.s003.docx]

**Defining the *in-vivo* phenotype of artemisinin resistant *falciparum* malaria: A modelling approach**

**Supporting information 3 - simulation of day 1, 2 and 3 percentage positive observations**

Day 1

A plot of the simulation model prediction for the relationship between the percentage of patients positive on day-1 of treatment and the percentage of resistant infections in the sample (solid black lines) with 95% prediction interval (dashed black lines). This prediction is plotted with a 90% threshold for the percentage of patients positive on day-1 of treatment (red dashed line). The rows of the panel represent three different assumptions about the mean parasitaemia on admission and the columns of the panel represent three different assumptions about the study sample size.

Day 2

A plot of the simulation model prediction for the relationship between the percentage of patients positive on day-2 of treatment and the percentage of resistant infections in the sample (solid black lines) with 95% prediction interval (dashed black lines). This prediction is plotted with a 30% threshold for the percentage of patients positive on day-2 of treatment (red dashed line). The rows of the panel represent three different assumptions about the mean parasitaemia on admission and the columns of the panel represent three different assumptions about the study sample size.

Day 3

A plot of the simulation model prediction for the relationship between the percentage of patients positive on day-3 of treatment and the percentage of resistant infections in the sample (solid black lines) with 95% prediction interval (dashed black lines). This prediction is plotted with a 10% threshold for the percentage of patients positive on day-3 of treatment (red dashed line). The rows of the panel represent three different assumptions about the mean parasitaemia on admission and the columns of the panel represent three different assumptions about the study sample size.
